# Characterization of EGF-guided MDA-MB-231 cell chemotaxis *in vitro* using a physiological and highly sensitive assay system

**DOI:** 10.1371/journal.pone.0203040

**Published:** 2018-09-13

**Authors:** Verena Biswenger, Nina Baumann, Johannes Jürschick, Martina Häckl, Christopher Battle, Jan Schwarz, Elias Horn, Roman Zantl

**Affiliations:** 1 ibidi GmbH, Martinsried, Germany; 2 Hochschule Weihenstephan-Triesdorf, Freising, Germany; Universita degli Studi di Parma, ITALY

## Abstract

Chemotactic cell migration is a central mechanism during cancer cell invasion and hence metastasis. In order to mimic *in vivo* conditions, we used a three-dimensional hydrogel matrix made of collagen I and a stable gradient-generating chemotaxis assay system, which is commercially available (μ-Slide Chemotaxis) to characterize epidermal growth factor (EGF)-induced chemotaxis of the human breast cancer cell line MDA-MB-231. Surprisingly, chemotactic effects of EGF on MDA-MB-231 cells could neither be observed in the standard growth medium DMEM/F-12 supplemented with 10% serum nor in starvation medium. In contrast, after adapting the cells to the serum-free growth medium UltraCULTURE^TM^, significant chemotactic effects could be measured with high sensitivity. The extremely time-stable linear gradients, generated in the chemotaxis chamber, led to consistent directional migration of MDA-MB-231 cells. Dose-response experiments showed increased directional and kinetic response of MDA-MB-231 cells towards stable gradients of EGF. While EGF-guided directional migration (chemotaxis) was highly concentration-dependent with the highest response at 1.5 nM/mm EGF, we found that the chemokinetic effect induced by EGF was concentration-independent. Both, blocking the ligand-binding domain of the EGF receptor by an antibody (monoclonal anti-EGFR antibody 225) and inhibition of its kinase domain by a small molecule inhibitor (AG1478) led to a reduction in EGF-induced directed migration. The high sensitivity of the assay even allowed us to observe synergistic effects in EGF-receptor inhibition using a combination of low doses of both inhibitor types. Those results validate the fact that EGF is a potent guidance cue for MDA-MB-231 cell migration and help to understand the mechanism behind chemotaxis-driven cancer metastasis.

## Introduction

Chemotactic cell migration, the directional orientation of a cell in response to extracellular chemical guidance cues, has been in focus of research for more than a century due to its involvement in several important physiological and pathological processes such as angiogenesis [1, 2], inflammation [3], tumor growth, and metastasis [4, 5]. To successfully metastasize, a carcinoma cell must invade, intravasate, transit in the blood or lymph, extravasate, and grow at a distant site [6]. Hereby, chemotaxis is thought to be involved in each of these crucial steps of tumor cell dissemination [4, 5, 7] with chemokines and growth factors being identified as potent guidance cues.

One particular molecular target of high promise in oncology is the epidermal growth factor (EGF) and its receptor (EGFR), since it has been found to be overexpressed, dysregulated, or mutated in many epithelial malignancies [8–11].

Growth factor receptors, like EGFR, belong to the family of receptor tyrosine kinases that contribute to complex signaling cascades modulating growth, signaling, differentiation, adhesion, migration, and survival of cancer cells. The receptors contain an extracellular ligand-binding domain, a hydrophobic transmembrane region and a cytoplasmic tyrosine kinase domain, which is activated by receptor dimerization upon growth factor binding [11, 12]. Two distinct therapeutic approaches are currently employed for targeting EGFR [8–10]. Firstly, there are monoclonal antibodies (mAbs) specifically designed to be directed against the extracellular domain, thus blocking ligand binding. Consequently, receptor dimerization, auto-phosphorylation and downstream signaling are prevented. Tyrosine kinase inhibitors (TKIs) are used in the second approach. They compete for intracellular kinase domain binding with adenosine triphosphate (ATP) and thus diminish downstream signaling. Several candidates of both groups or combinations were tested *in vitro*, in animal models, or reached various stages of clinical development [8, 13].

Despite its clinical importance, relatively little is known about the underlying mechanisms of EGF-guided tumor cell chemoinvasion *in vivo*. The behavior of cancer cells is influenced by an interaction of multiple factors such as the communication between different cell types, the microenvironment such as the presence of different molecules and potential guidance cues, like growth factors, as well as their presentation [4, 14, 15].

Thus, to improve our understanding of how multiple exogenous factors affect tumor cell motility and chemoinvasion, robust *in vitro* models are necessary. These models should be designed to fulfil the following requirements: (i) offering physiological relevant 3D conditions, (ii) presentation of stable and robust gradients of guidance cue, and (iii) allow for a detailed analysis on a single cell level. However, many of the commonly used models, such as transwell based systems, have limitations and do not meet all of these requirements. Recently, several additional techniques became popular for measuring chemotaxis *in vitro*, such as microfluidic devices, allowing to create more physiologically relevant model systems. However, those applications are very complex as they require flow-producing equipment [16–18]. Here, we use an easy to handle diffusion-based chemotaxis system that meets the above mentioned requirements.

Apart from the technical requirements, the medium composition used for cell cultivation before and during the experiment can have an impact on the fitness of cells and thus on the assay sensitivity [19, 20]. Usually, basal cell culture medium is supplemented with animal serum. Serum is a complex mixture of different factors and contains a large number of components, such as growth factors, proteins, vitamins, trace elements, hormones, etc., which are essential for cell attachment, growth and proliferation [21, 22]. Its composition is not known in detail, thus introducing an undefined variable into the culture system. Furthermore, seasonal and continental differences in the serum composition produce batch-to-batch variation, limiting the reproducibility of performed experiments. To overcome these limitations and to increase the reproducibility, serum-free growth medium with a defined composition is available.

In order to analyze EGF-induced chemotaxis of MDA-MB-231 cells in a highly reproducible and sensitive manner, we used a commercially available chemotaxis chamber. The geometry of the chamber allowed cell migration under physiological 3D conditions, the formation of a time-stable and robust chemical gradient, as well as time-lapse microscopy. The latter formed the basis for the detailed single cell analysis of the migratory behavior of MDA-MB-231 cells. Additionally, adapting MDA-MB-231 cells to a serum-free growth medium allowed us to significantly increase the sensitivity of the model. Hence, we were able to characterize EGF-mediated chemotaxis of MDA-MB-231 cells in a very detailed manner, including dose-dependency analysis and specific inhibition of EGF-guided migration using different inhibitor types. Furthermore, the high sensitivity of the model allowed us to observe synergistic effects in EGFR inhibition using a combination of low doses of different inhibitor types.

## Materials and methods

### Fluorescence correlation spectroscopy

Fluorescence correlation spectroscopy measurements were performed using an inverted confocal Axiovert 200/LSM 510 microscope (Carl Zeiss) equipped with a 488 nm argon laser and standard Zeiss filter set and controlled with the ImageJ software plugin Micro-Manager (Ron Vale, University of California). AlexaFluor 488 (Invitrogen) concentration profiles with C_0_ = 1 nM and C_100_ = 50 nM in 1x PBS (Sigma-Aldrich) were generated in a 1.5 mg/ml bovine collagen type I (PureCol, Advanced BioMatrix) gel. Fluorescence intensities were measured at 22 °C in the middle of the observation area (y-position), 35 μm above the bottom (z-position), and at five different x-positions (–500 μm, –250 μm, 0 μm, 250 μm, and 500 μm).

### Cell culture

MDA-MB-231 cells (DSMZ, DSMZ no. ACC-732) were maintained in the basal medium Dulbecco’s Modified Eagle`s Medium/Nutrient Mixture F-12 Ham (DMEM/F-12; Sigma-Aldrich) supplemented with 10% fetal calf serum (FCS; Sigma-Aldrich) at 37 °C in a humidified incubator at 5% CO_2_. Cells were subcultured after reaching an optical confluence of 70–90%. Cells were adapted to the serum-free growth media UltraCULTURE^TM^ (UC; Lonza) and PanSerin 401 (PS; PAN-Biotech) by lowering the content of the original serum-containing medium in a stepwise fashion. The adaptation was performed in five subcultivation steps, replacing each time 20% serum-containing base medium by UC or PS. Cell culture flasks were coated with bovine collagen type I (PureCol, Advanced BioMatrix).

### Chemotaxis assay

Cell migration assays were performed using the μ-Slide Chemotaxis (ibidi GmbH) according to the manufacturer’s protocol. To obtain a 1.5 mg/ml gel solution, 150 μl bovine collagen type I (PureCol, Advanced BioMatrix) was mixed with 20 μl 10x DMEM (Sigma-Aldrich), 6 μl 1 M NaOH (Carl Roth), 14 μl H_2_O (Carl Roth), 10 μl 7.5% NaHCO_3_ (Sigma-Aldrich), 50 μl medium with respective supplements, and 50 μl cell suspension. Cell suspension and gel mixture were mixed yielding a final cell concentration of 2x10^6^ cells/ml and loaded into the migration chamber. After gelation, reservoirs were filled either with medium containing chemoattractant or neutral medium. EGF (PromoCell) was used as chemoattractant, pure serum-free UC or DMEM/F-12 containing different FCS concentrations as neutral medium. The following experiments were performed: a negative control (indicated as -/-) with no chemoattractant; a positive control (indicated as +/+) with both reservoirs and the gel solution containing chemoattractant; and an experimental group (indicated as +/-) where only one chamber was filled with chemoattractant. For inhibition of EGFR, AG1478 (Sigma-Aldrich), EGFR Monoclonal Antibody 225 (ThermoFisher Scientific), or a combination of both was added to the entire chamber system to avoid potential inhibitor gradient formation.

The assay was independently repeated at least three times including technical replicates. Data are expressed as mean and error bars define the standard error of the mean (SEM).

### Cell viability studies

Cell viability studies were performed using a fluorescein diacetate (FDA; Sigma-Aldrich) and propidium iodide (PI; Sigma-Aldrich) live/dead staining. For the staining solution, 5 ml culture medium without FCS were mixed with 8 μl FDA (5 mg/ml) and 50 μl PI (2 mg/ml). After 24 hours, cell culture medium was removed from the reservoirs of the chemotaxis chamber and reservoirs were refilled with staining solution. After 5 minutes at room temperature, staining solution was removed and reservoirs were refilled with 1x PBS. Samples were analyzed using the inverted microscope Nikon Eclipse Ti equipped with a CCD camera Orca-Flash 4.0 LT (Hamamatsu) and the appropriate filter sets. The ImageJ software plugin “Micro-Manager” (Ron Vale, University of California) was used for image acquisition. The number of dead (PI positive) and living cells (FDA positive) was manually counted to calculate the cell viability using the ImageJ software.

### Time-lapse microscopy and migration data analysis

Time-lapse microscopy was performed using the inverted microscopes Nikon Eclipse Ti equipped with a CCD camera Orca-Flash 4.0 LT (Hamamatsu) and Olympus CKX 41 equipped with a CCD camera SC30 (Olympus). For image acquisition, the ImageJ software plugin “Micro-Manager” (Ron Vale, University of California) was used at the Nikon microscope and the cellSens Dimension software (Olympus) at the Olympus microscope. Both microscopes were equipped with the ibidi Heating System (37 °C) and the ibidi Gas Incubation System (actively mixed 5% CO_2_ at a flow rate of 10 l/hour and 90% humidity).

An interval of 10 minutes was used to perform time-lapse microscopy over a period of 24 hours. Cell tracking was performed using the ImageJ software plugin “Manual Tracking” (Fabrice Cordelières, Institut Curie). 40 cells per experiment were manually tracked and the directionality and speed of each single cell analyzed using the ImageJ software plugin “Chemotaxis and Migration Tool V2.0” (ibidi GmbH) according to the instructions of the manufacturer. Briefly, directionalities were evaluated as forward migration indices in direction of the gradient (FMI^II^) and perpendicular to the concentration gradient (FMI┴). As a first step, cell trajectories were extrapolated to (x,y) = 0, at time point 0 h (= slice 0) (S1 Fig). FMIs were then calculated as follows:

$${FMI}^{┴}=\frac{1}{n}\sum_{i=1}^{n}\frac{x_{i,end}}{d_{i,accum}}$$

$${FMI}^{II}=\frac{1}{n}\sum_{i=1}^{n}\frac{y_{i,end}}{d_{i,accum}}$$

n = number of cells

x_i,end_, y_i,end_ = coordinates of cell end point

d_i,accum_ = accumulated distance of cell path

A detailed description of all parameters generated by the Chemotaxis and Migration Tool is published by Zengel et al. [23].

To compare different conditions, all single cell FMI values were averaged. For statistically relevant chemotactic effects, the averaged absolute FMI^II^ values of the experimental group must be significantly higher than the averaged FMI^II^ values of the respective positive and negative controls, and significantly higher than the corresponding averaged FMI┴ values. Cell speed was used to evaluate changes in cell motility and random cell migration.

### Determination of dissociation constant K_d_

The velocity data of all cells in direction of the EGF gradient (V_II_) was calculated and the biologically relevant part was fitted to the model described in Haessler et al. [16] to determine the dissociation constant (K_d_) of EGF. The model was described as follows:

$$V_{II}=A*\frac{\nabla c}{{\left(c_{avg}+K_{d}\right)}^{2}}$$

V_II_ = average cell velocity in direction of the gradient

A = constant

∇c = absolute concentration gradient

c_avg_ = average concentration

K_d_ = dissociation constant

The model was fitted using the nls package in R with weighted non-linear least squares regression. The constant A and K_d_ were fitted by the model as well as an additional offset degree of freedom. Error bars represent the standard error of the mean of the average cell velocity (V_II_) at each concentration gradient.

### Statistical analysis

For statistical analysis, an ordinary one-way analysis of variance (ANOVA) test was performed with Tukey’s correction for multiple comparisons using GraphPad Prism (GraphPad Software). Statistical significances of each experiment are given in the respective figure legends.

## Results and discussion

### Experimental setup and migration analysis

**Fig 1:**
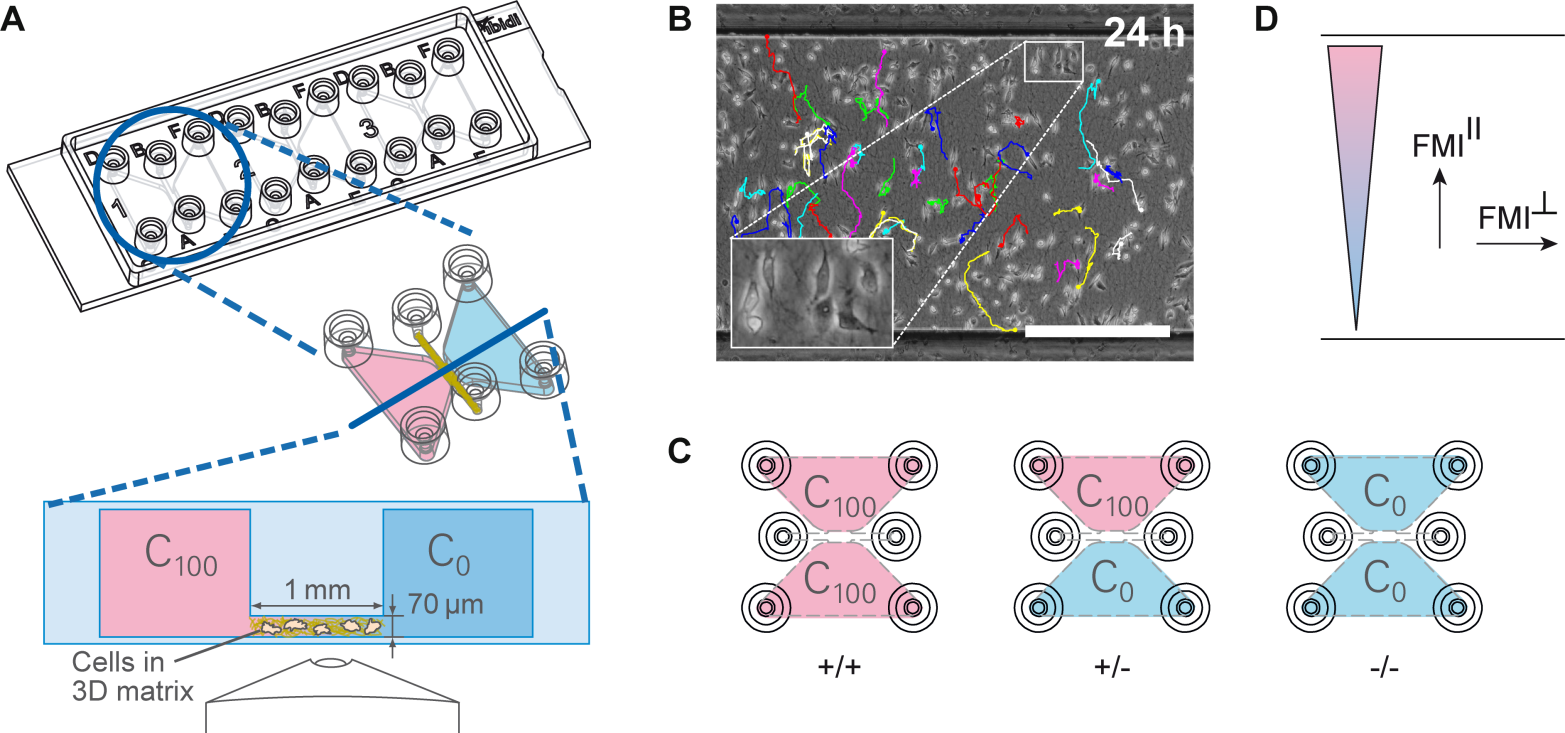
Experimental setup and migration analysis. (A) Technical drawing of the **μ**-Slide Chemotaxis showing the three adjacent chambers for parallel experiments. Two reservoirs are connected by a central observation area (height: 70 **μ**m, width: 1 mm). Single cells are embedded in a 3D matrix inside the observation area. A time-stable gradient can be established by filling the reservoirs with different concentrations of chemoattractant (C_0_ and C_100_). (B) Representative image of the observation area with MDA-MB-231 cells embedded in a collagen gel with an overlay of the manually tracked cell trajectories at time point 24 h. Scale bar represents 500 **μ**m. (C) Each experimental setup includes three conditions: the experimental condition offering a concentration gradient (+/-) and the two controls with chemoattractant being present in the entire system (+/+) or without chemoattractant (-/-). (D) Analysis of chemotaxis by forward migration indices in direction of the gradient (FMI^II^) and perpendicular to the gradient (FMI┴). FMI^II^ values are high for directional migration and close to zero for random migration.

The migration of tumor cells is strongly determined by their physical and chemical microenvironment [4, 14, 15]. This microenvironment consists of different cell types, a diversity of growth factors and cytokines, as well as the surrounding extracellular matrix. Transwell and wound healing assays are commonly used assays to assess the migration of cancer cells in response to external stimuli. These assays lack several essential elements of the tumor microenvironment, such as the 3D extracellular matrix. Hence, in order to study the chemotactic behavior of MDA-MB-231 cells in a physiological relevant 3D environment, we performed chemotaxis experiments using a commercially available chemotaxis chamber (μ-Slide Chemotaxis, ibidi GmbH) and a 1.5 mg/ml bovine collagen type I gel-matrix. Type I collagen is the main component of connective tissue and offers an optimal cell migration at 1.5 mg/ml [16, 24–26]. The system contains three adjacent chambers to enable parallel assays of different migration conditions (Fig 1A, top). Each chamber consists of two large reservoirs that are connected by a narrow observation area (Fig 1A, bottom). Cells are embedded in the gel-matrix inside the observation area and can be super-imposed by a linear and time-stable gradient. The presence of this time-stable gradient allows long-term observation of cellular migratory behavior. We used time-lapse microscopy and manual tracking of single cells to generate single-cell trajectories (Fig 1B) which were analyzed for chemotactic and chemokinetic effects.

Each experimental setup included three different conditions (Fig 1C): one experimental condition and two controls. The experimental condition offers a concentration gradient (+/-). As a control, chemoattractant is either added to the entire system (+/+) or is absent in the entire system (-/-). These controls help to distinguish between directed migration and arbitrary migration effects, like enhanced motility.

In order to analyze the directionality of cell migration, averaged forward migration indices (FMIs) were calculated, as shown in the materials and methods section [23]. We distinguish between FMI values in direction of the concentration gradient (FMI^II^) and perpendicular to the concentration gradient (FMI┴) to evaluate chemotaxis (Fig 1D) [23]. Random cell migration exhibits FMI^II^ and FMI┴ values close to zero, when averaged over all single cells. For directed migration in direction of the gradient, the averaged FMI^II^ values of the experimental group must be significantly higher than the averaged FMI^II^ values of the respective positive (+/+) and negative controls (-/-). Additionally, the FMI┴ values averaged over all single cells of the experimental group need to be close to zero to exclude biased migration by non-chemotactically environmental factors.

Mammalian cells can migrate along the extracellular matrix scaffold [17, 27]. Therefore, scaffold orientation might induce a directional bias for cell migration dependent on adhesion to the scaffold. Due to handling and polymerization effects, the fibers of collagen gel matrices *in vitro* might be oriented in a specific direction. This fiber orientation might in turn lead to biased migration of migrating MDA-MB-231 cells in *in vitro* assays, which can be identified by increased FMI┴ values. Typically, fiber orientations in the chemotaxis chamber are randomly distributed, resulting in FMI┴ values close to zero.

### Time-stable linear gradients within the chemotaxis chamber

Migrating MDA-MB-231 cells exhibit a considerably slower average migration speed than e.g. leukocytes [27]. Thus, to observe chemotactic effects for MDA-MB-231 cells, gradients of the guidance cue need to be defined and presumably stable over an extended period of time [23].

**Fig 2:**
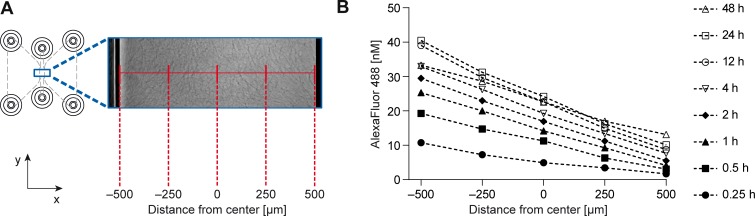
Time-stable linear concentration profiles created within the migration chamber. (A) Top view of the chemotaxis chamber with a collagen I matrix in the observation area, one reservoir filled with 50 nM of AlexaFluor 488 and the other reservoir filled with 1 nM of AlexaFluor 488. Measurements were performed in the middle of the observation area (y-position), 35 **μ**m above the bottom (z-position) and at five different x-positions, indicated in red. (B) Time dependence of the AlexaFluor 488 concentration profile, measured in a collagen I gel by fluorescence correlation spectroscopy.

In order to characterize the formation kinetics and properties of the gradients generated within the collagen gel of the migration chamber, fluorescence correlation spectroscopy measurements were performed (Fig 2A). A shallow gradient could already be detected 15 minutes after filling the reservoirs with fluorophore solution. After approximately 4 hours, a stable linear gradient could be observed that reached steady-state and lasted at least 48 hours without detectable changes in the concentration distribution (Fig 2B). Those results show that the chambers offer defined and time-stable gradients, thus allowed the performance of reliable and reproducible chemotaxis assays.

### Successful adaptation and maintenance of MDA-MB-231 cells in serum-free growth medium

Almost every cell type has its own medium and supplement requirements. Cell culture medium must supply all essential nutrients for cell metabolism, growth, and proliferation. DMEM/F-12 medium is a non-supplemented basal medium, lacking special nutrients. Adding serum to the basal medium is an almost universal growth supplement, which is effective for most types of human and animal cells as it contains most of the factors required for cell proliferation and maintenance [21]. However, the usage of animal serum for cell cultivation before and during experiments introduces an undefined variable to experimental setups. Guidance cues, for example, which are present in varying concentrations in animal serum, might reduce the reliability and reproducibility of migration assays performed in the presence of serum. Therefore, serum-free growth media are available. They are complete, allpurpose media supplemented with cell-type specific components and designed for the cultivation of a wide variety of adherent and non-adherent mammalian cell types.

In order to increase the sensitivity and the accuracy of the migration assay without a reduction in cell viability, we tested whether MDA-MB-231 cells could be cultivated and assayed in serum-free growth medium. We tested two different serum-free growth media for fibroblasts, UltraCULTURE^TM^ (UC) and PanSerin 401 (PS). UC is based on the basal medium DMEM/F-12 and PS on Iscove’s MEM and both are supplemented with defined serum components and nutrients.

Reducing the serum content in the mixture of PS and DMEM/F-12 below 1% (90% PS, 10% DMEM/F-12 supplemented with 10% serum) resulted in reduced cell adherence, reduced cell proliferation and changes in morphology. Hence, MDA-MB-231 cells could not be adapted to the serum-free growth medium PS. In contrast, MDA-MB-231 cells could be successfully adapted to and maintained in the serum-free growth medium UC. Growth kinetics, such as the doubling time (27.5 hours for UC), were comparable to MDA-MB-231 cells cultivated with 10% serum-containing basal medium DMEM/F-12 (28.1 hours).

The reason why MDA-MB-231 cells could be successfully adapted to the serum-free growth medium UC, but not to PS might be the different supplement composition of both growth media. According to the manufacturer, the medium UC consists of a DMEM/F-12 base supplemented with recombinant human insulin, bovine transferrin and a purified mixture of bovine serum proteins including albumin. PS, by contrast, consists of an Iscove’s MEM base supplemented with trace elements, albumin, cholesterol, soy-lipids and vitamins. Notably, PS does not contain any growth or attachment factors. Our findings suggest that the composition of PS is not optimal for MDA-MB-231 cells, resulting in reduced cell adherence and proliferation as well as changes in cell morphology. Consequently, MDA-MB-231 cells cultivated in UC were used for the migration studies.

### Enhanced chemotactic response of MDA-MB-231 cells in serum-free medium

Not only varying serum composition, but also being permanently exposed to the large number of different factors present in serum might have an influence on the sensitivity of cells, for example due to enzyme saturation [28], enzyme conformation changes [29], or desensitization effects [30, 31]. Therefore, many cell based assays, such as receptor activation, proliferation, or migration assays are performed with serum-starved cells, even though little is known about the possible altered responses due to the starvation conditions [32]. In order to quantify the influence of serum on the cellular fitness and the chemotactic response, we analyzed viability and EGF-guided directional migration of MDA-MB-231 cells in the basal medium DMEM/F-12 at different serum contents and in the serum-free growth medium UC.

**Fig 3:**
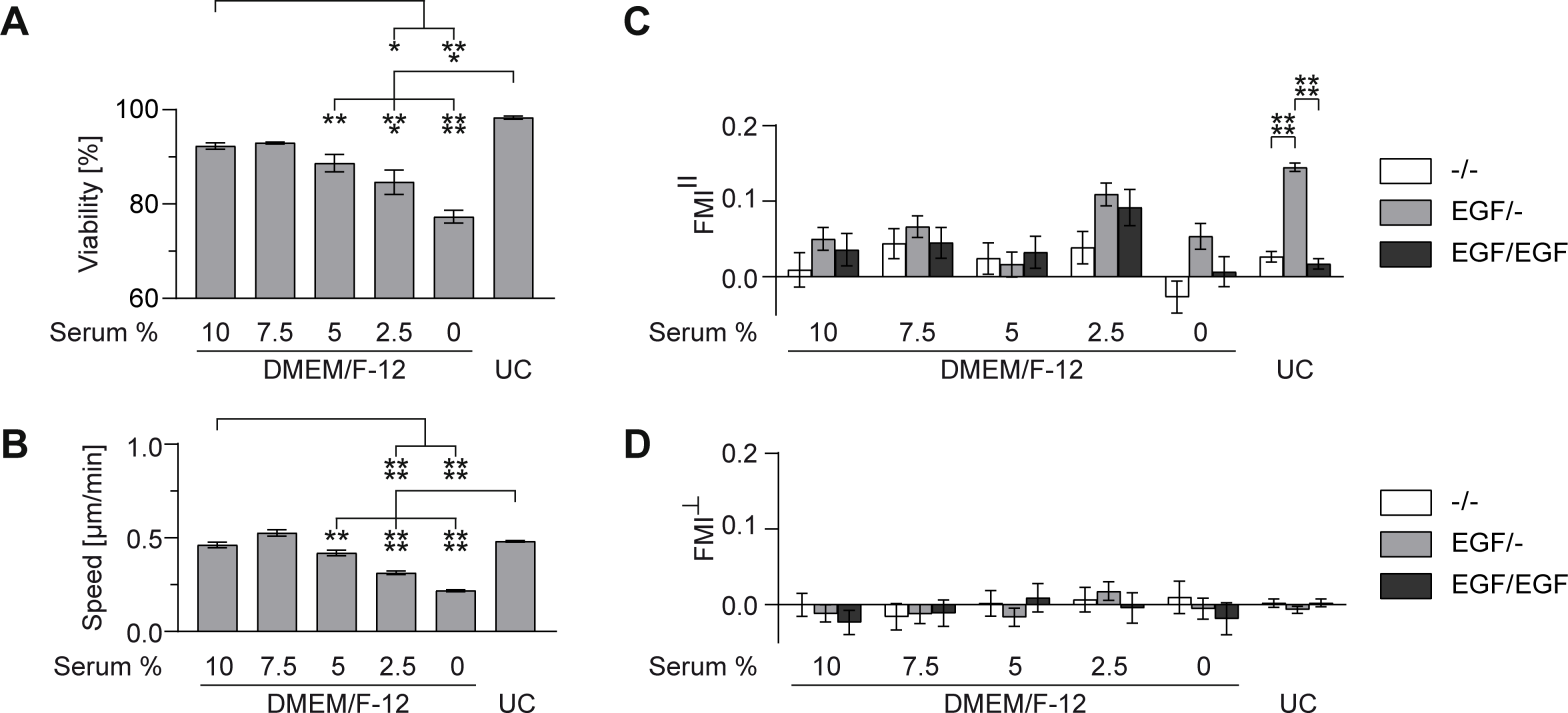
MDA-MB-231 cellular fitness and migration in standard DMEM/F-12 medium with different serum concentrations in comparison with serum-free growth medium UC. (A) Viability of MDA-MB-231 cells in chemoattractant-free medium. (B) Cell speed of MDA-MB-231 cells in chemoattractant-free medium. (C) Forward migration indices in direction of the gradient (FMI^II^) and (D) perpendicular to the gradient (FMI**┴**) of MDA-MB-231 cells migrating in pure medium (-/-), in a 1.5 nM/mm EGF gradient (EGF/-) and in 1.5 nM EGF in the entire chamber (EGF/EGF). Significances are indicated by asterisks with * for 0.01<p<0.05, ** for 0.001<p<0.01, *** for 0.0001<p<0.001, and **** for p<0.0001.

First, we analyzed whether serum reduction in the basal medium DMEM/F-12 or the usage of serum-free growth medium had an influence on the fitness of cells embedded in a collagen I gel inside the migration chamber. Therefore, the cell viability and speed were evaluated in the absence of any chemoattractant (Fig 3A and 3B). For quantification of cell viability, a live/dead staining was performed inside the chemotaxis chamber. Cell speed was determined by analyzing random cell migration.

We were able to observe comparably high cell viabilities (Fig 3A) in serum-free growth medium and the standard basal culture medium DMEM/F-12 supplemented with 10% serum as well as a similar cell speed (Fig 3B). However, serum reduction in the basal medium DMEM/F-12 resulted in a significant decrease in viability (Fig 3A) and cell speed (Fig 3B) in comparison to the basal medium supplemented with 10% serum and UC. The decrease in cell speed due to serum reduction could also be seen in the presence of EGF, regardless if applied as concentration gradient (EGF/-), or in the entire system (EGF/EGF, S2 Fig).

In order to test whether serum reduction in the basal medium DMEM/F-12 or serum substitution using a serum-free growth medium had an influence on EGF-sensing of MDA-MB-231 cells and hence directed migration, we performed chemotaxis assays towards stable gradients of EGF (1.5 nM/mm EGF). For cells migrating in DMEM/F-12 supplemented with different serum concentrations, the FMI^II^ values of the chemotaxis experiments were not significantly different from the control experiments, that were either performed in the absence (-/-) or presence of EGF (EGF/EGF) in the entire system. Hence, we were not able to observe significant EGF-induced chemotactic effects for MDA-MB-231 cells in presence of serum (Fig 3C). In contrast, experiments performed with serum-free growth medium UC revealed significant chemotactic migration of MDA-MB-231 cells towards stable gradients of EGF. FMI^II^ values (0.15±0.01 for EGF/-) were significantly different from the FMI^II^ values of the controls (0.03±0.01 for -/-, 0.02±0.01 for EGF/EGF) (Fig 3C). In addition, the FMI┴ values of all conditions were close to zero (Fig 3D).

Our findings revealed that MDA-MB-231 cells cultivated in the serum-free growth medium UC showed an increased chemotactic sensitivity towards the guidance cue EGF. No chemotactic effect could be observed for MDA-MB-231 cells cultivated in the basal medium DMEM/F-12 supplemented with 10% FCS. Furthermore, serum reduction in the basal medium DMEM/F-12 did not lead to an increased chemotactic sensitivity of MDA-MB-231 cells towards EGF. Serum reduction even resulted in reduced cell viability and cell speed over an assay period of 24 hours. These findings underpin the fact that a defined growth system is needed to obtain reliable results. Solely starving the cells might lead to biased migration effects and might also influence other cellular processes, resulting in altered migration behavior independent of the guidance cue.

**Fig 4:**
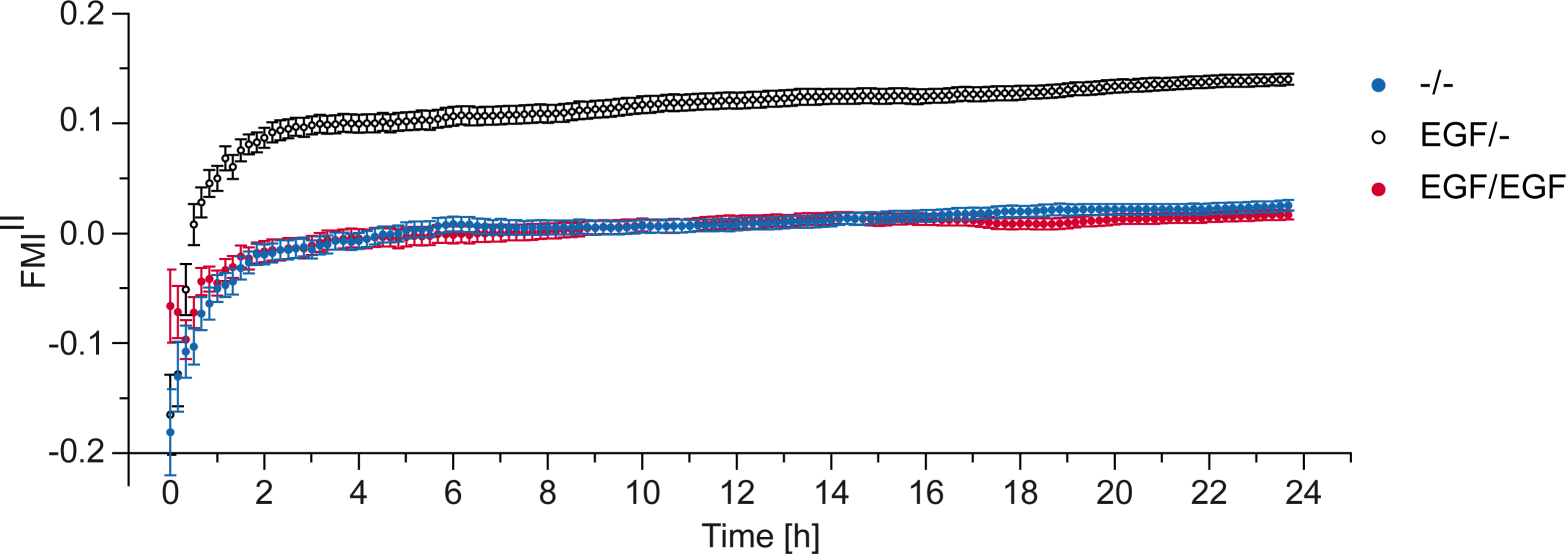
Time dependency of FMI^II^ values. Averaged FMI^II^ values of gradient experiment (EGF/-) and respective negative (-/-) and positive (EGF/EGF) control, plotted over time. As an example, 1.5 nM/mm EGF was used as gradient experiment.

To further characterize the migration behavior of MDA-MB-231 cells towards EGF, we performed a time-resolved directionality analysis (FMI^II^) in UC medium (Fig 4). For each time point, the average FMI^II^ over all single cells recorded in the experiment was calculated. This analysis showed constant average FMI^II^ values of about 0.15±0.01 after an initial built-up phase of 2 hours. The built-up phase can be explained by a statistical effect rather than a biological one, as cell trajectories do not confirm this “ramping” behavior (S3A and S3B Fig). Depending on the accumulated distance a cell is covering, the calculated FMI is spreading for short cell trajectories and stabilizes for longer accumulated distances. To proof this assumption we performed cell migration simulations (random walk and biased random walk). The directionality analysis of those simulated trajectories showed the same FMI spreading for short cell trajectories (S3E Fig). Additionally, as expected the FMI^II^ values of the control experiments (-/-, EGF/EGF) adjust close to zero after a similar built-up phase. The stable FMI^II^ values are in line with the time-stable linear concentration gradients measured in the chemotaxis chamber (Fig 2B). Therefore, we conclude that MDA-MB-231 cells are able to sense stable, linear gradients of EGF, as they induced a stable migration behavior. In addition, MDA-MB-231 cells do not show EGF-induced desensitization during the time-course of our experiments. Furthermore, time-stable gradients, generated in the migration chambers and the induced stable chemotactic migration, are necessary to obtain statistically robust data, which allows the characterization of even small chemotactic effects.

### Dose-dependency of EGF-induced chemotaxis

EGF is an important growth factor, which has been associated with tumor progression. Using transwell migration assays, Sun et al. [33] and Price et al. [34] showed EGF induced trans-migration of MDA-MB-231 cells and were therefore able to describe EGF as potent inducer of MDA-MB-231 cancer cell migration. The results obtained using transwell migration assays are a superposition of three effects: (1) directed migration, (2) increase in cell motility, and (3) adhesion of cells to the membrane surface. Consequently, these systems failed to distinguish between chemotactic or chemokinetic EGF guidance across the membrane of the chamber. Additionally, the usage of the transwell system prohibited any characterization of EGF gradient properties. Surprisingly, using a diffusion based microfluidic setup, which allowed to characterize migration induced by defined linear gradients of EGF, Kim et al. [17] and Wang et al. [18] were not able to observe any EGF induced directional response of MDA-MB-231 cells. Therefore, we intended to use our diffusion based sensitive assay system in combination with serum-free UC medium to characterize EGF-guided MDA-MB-231 cancer cell migration and to dissect chemotactic from chemokinetic behavior.

**Fig 5:**
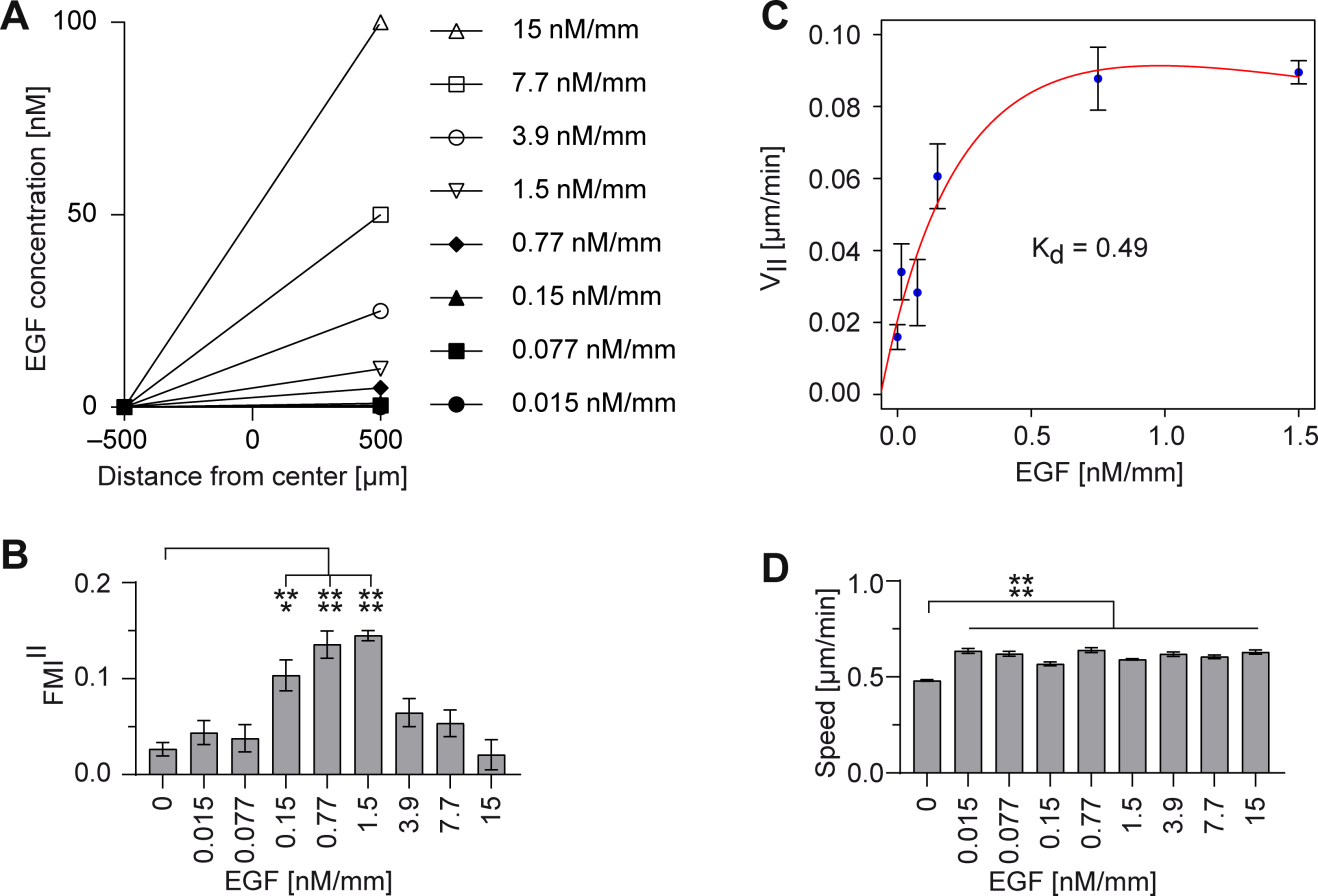
MDA-MB-231 cell migration in linear EGF gradients. Serum-free medium containing EGF in different concentrations (0.015–15 nM) was filled in one reservoir and pure serum-free medium in the other reservoir (EGF/-). (A) MDA-MB-231 cells were exposed to time-stable linear gradients of EGF (0.015–15 nM/mm). Cell migration was evaluated by (B) the forward migration indices in direction of the gradient (FMI^II^) and (D) the cell speed. (C) Cell velocity in direction of the gradient (V_II_) was used to calculate the dissociation constant K_d_. Significances are indicated by asterisks with * for 0.01<p<0.05, ** for 0.001<p<0.01, *** for 0.0001<p<0.001, and **** for p<0.0001.

MDA-MB-231 cells were exposed to time-stable linear gradients of EGF with concentration gradients ranging from 0.015 to 15 nM/mm (Fig 5A). Subsequently, chemotactic and chemokinetic effects were characterized by analyzing the directionality (FMI^II^) and speed of migrating MDA-MB-231 cells.

Consistent with previous reports for transwell chemotaxis assays [33, 34], EGF induced chemotaxis in a concentration-dependent manner (Fig 5B). Increasing the EGF concentration led to an increase in chemotactic response of MDA-MB-231 cells, with the highest significant chemotaxis for an EGF concentration gradient of 1.5 nM/mm with FMI^II^ values of 0.15±0.01. However, the chemotactic response decreases with further increasing the EGF concentration. In addition, concentration gradients of EGF as low as 0.015 nM/mm, which did not induce any directional response (Fig 5B), already led to a significant increase in cell speed (Fig 5D). However, in contrast to the concentration dependency of the directional response, we were not able to detect dose-dependent differences for cell speed. Additionally, EGF-dependent, but dose-independent increase in speed could be detected regardless if applied as concentration gradient (EGF/-, Fig 5D), or in the entire system (EGF/EGF, S4 Fig). EGF-dependent increase in cell speed could even be assumed in serum-containing DMEM/F-12 medium, although not in a consistent manner (S2 Fig).

Those results confirm that MDA-MB-231 cells are able to interpret stable, diffusion based, linear gradients. MDA-MB-231 cells are able to sense EGF concentration gradients as low as 0.015 nM/mm, indicated by an increase in cell motility. However, concentration gradients of 0.15 to 1.5 nM/mm were needed to induce directional migration. Assumed, MDA-MB-231 cells detect concentration differences of guidance cues across the cell body [35], a sufficient difference in the concentration of external gradients across the cell is required to activate chemotaxis. As all tested concentration gradients are linear, only differing in their maximal concentration, they shared the same signal-to-noise relation ▿c/c (S5 Fig). Therefore, concentration-dependent differences in chemotactic response seem to be dependent on global EGF concentration available in the system. Thus, for an EGF gradient steepness higher than 1.5 nM/mm, a saturation effect can be observed. This effect might be explained by fully occupied EGFR or slow receptor desensitization. The measured chemokinetic effect for an EGF gradient steepness higher than 1.5 nM/mm, indicating ongoing EGFR signaling, supports this hypothesis (Fig 5D). In addition, similar findings for receptor desensitization can be found in the literature indicating desensitization at similar EGF concentrations [33, 34].

The narrow chemotactically effective EGF gradient ranges and the necessity of time-stable gradients could explain why other experimental setups failed to observe EGF-guided MDA-MB-231 chemotaxis. Furthermore, single cell analysis and extensive control conditions allowed us to distinguish between arbitrary migration effects, like enhanced motility and chemotaxis.

To evaluate the binding kinetics of EGF, cell velocity in EGF gradient direction (V_II_) was calculated and applied to a kinetic equation described by Haessler et al. [16]. Since the average cell velocity demonstrated saturation effects above 1.5 nM/mm, only concentration gradients between 0 nM/mm and 1.5 nM/mm were used to fit the dissociation constant K_d_ (Fig 5C). EGF binds to its receptor in MDA-MB-231 cells with a K_d_ value of approximately 0.49 nM over a channel width of 1 mm. The EGFR binding affinity has been reported several times in the literature and resulted in values ranging from 0.0177 nM (*in vitro* measurements) to 5 nM (binding assay in living cells) [36, 37]. Interestingly, Björkelund et al. investigated, that the affinity and kinetics of EGF binding to EGFR is greatly dependent on the cell line, with a K_d_ value of approximately 0.2 nM for breast cancer cells [38]. These findings comply with the K_d_ we determined for the breast cancer cell line MDA-MB-231.

Summarized, using our sensitive assay system, we could show that low concentrations of EGF induce a chemokinetic effect on MDA-MB-231 cells, however, failed to directionally guide the cells. Effective and significant guidance can be observed for EGF gradients with a steepness ranging from 0.15 to 1.5 nM/mm. Gradients offering a steepness higher or equal to 3.9 nM/mm most likely lead to receptor saturation and therefore, to reduced directionality. Apparently, saturated receptors are still capable to maintain high cell speed.

### EGF-mediated chemotaxis of MDA-MB-231 cells was validated by EGFR signaling inhibition

In order to validate EGF as a chemotactic guidance cue for MDA-MB-231, chemotaxis assays were performed in the presence of EGFR-specific inhibitors. Two different approaches are commonly used, which target EGFR and interfere with EGFR-mediated effects [9, 10]. These include monoclonal antibodies (mAbs) and small molecule tyrosine kinase inhibitors (TKI). Both inhibitor types share the same target, but display different mechanisms of action and different specificity for EGFR. mAbs bind to the extracellular domain of EGFR and compete with endogenous ligands for the ligand-binding region, which results in blocking of the ligand-induced EGFR signaling, induced by EGFR activation. Small molecule TKIs compete with adenosine triphosphate to bind to the intracellular catalytic tyrosine kinase domain of EGFR, thereby inhibiting the autophosphorylation and downstream signaling.

To evaluate the effect on EGFR signaling and chemotaxis, the TKI AG1478 and the monoclonal anti-EGFR antibody 225 (mAb) were applied at different concentrations. Inhibitors were added to the entire system to prevent the formation of an inhibitor gradient. Directionality of cell migration (FMI^II^ in gradient direction) and speed were measured to evaluate inhibition effects. An EGF gradient steepness of 1.5 nM/mm was used, as this concentration gradient induced the highest chemotactic response of MDA-MB-231 cells with FMI^II^ values of 0.15±0.01 and cell speeds of 0.59±0.01 μm/min. A negative control without EGF in the entire system (ctrl) was used as reference for successful inhibition with FMI^II^ values of 0.03±0.01 and cell speeds of 0.48±0.01 μm/min.

**Fig 6:**
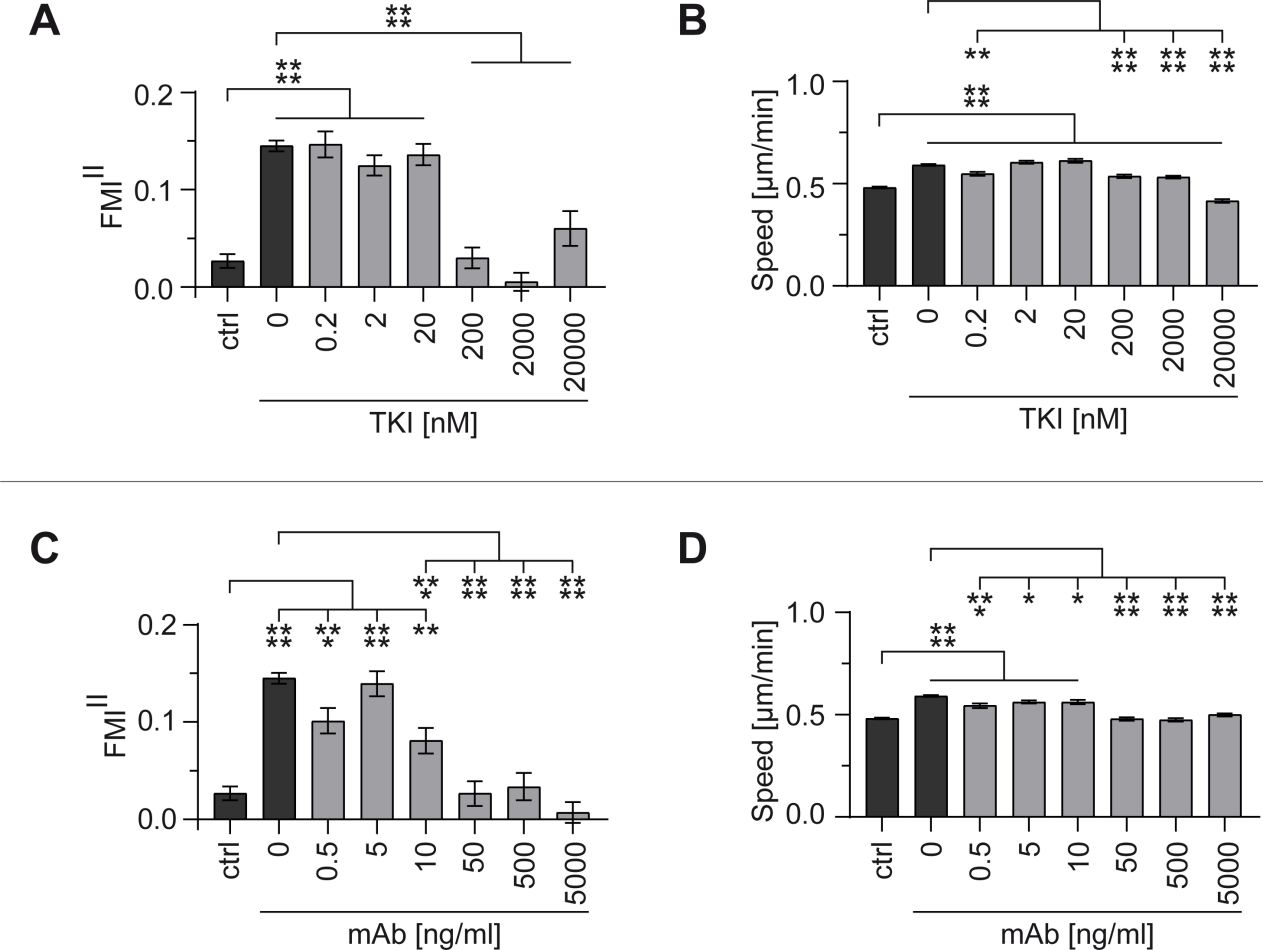
Inhibition of MDA-MB-231 cell migration in a 1.5 nM/mm EGF gradient. The effect of different EGFR inhibitors on migration was evaluated by the forward migration indices in direction of the gradient (FMI^II^) and the cell speed, and compared to the respective negative control without EGF gradient (ctrl). (A) FMI^II^ and (B) speed using tyrosine kinase inhibitor AG1478 (as TKI) (0.2 nM–20 **μ**M). (C) FMI^II^ and (D) speed using monoclonal anti-EGFR antibody (as mAb) (0.5 ng/ml–5 **μ**g/ml). Significances are indicated by asterisks with * for 0.01<p<0.05, ** for 0.001<p<0.01, *** for 0.0001<p<0.001, and **** for p<0.0001.

Using the TKI AG1478, directed cell migration was inhibited in a dose-dependent manner (Fig 6A). TKI concentrations of up to 20 nM did not have any effect on directional sensing and cell speed (Fig 6A and 6B). However, for TKI concentrations higher or equal to 200 nM complete inhibition of chemotaxis could be observed, with FMI^II^ values comparable to samples without EGF gradient (ctrl). Significant inhibition of chemokinetic effects could only be observed for high TKI concentrations (200–20,000 nM). Cell speed was even reduced below control level for a very high TKI concentration (20,000 nM).

Similar dose-dependent inhibition characteristics of chemotaxis could be observed for the monoclonal anti-EGFR antibody (Fig 6C). mAb concentrations of up to 10 ng/ml did not have any effect on directional sensing and cell speed (Fig 6C and 6D). However, for mAb concentrations higher or equal to 50 ng/ml complete inhibition of chemotaxis could be observed, with FMI^II^ values comparable to samples without EGF gradient (ctrl). Furthermore, these concentrations significantly inhibited the chemokinetic effects.

Our findings show that the observed directed migration of MDA-MB-231 cells and the increased chemokinesis were triggered by the addition of the guidance cue EGF. Both inhibitors blocked EGF-mediated chemotaxis in a dose-dependent manner, even though they display different mechanisms of action.

### The combination of two different inhibition strategies showed synergistic inhibition of EGFR already at low inhibitor concentrations

Our results show that high inhibitor concentrations are needed to significantly reduce the chemotaxis of MDA-MB-231 cells towards EGF when using both inhibitor types separately. Because the two classes of inhibitors target EGFR at different sites, we exploited the high sensitivity of our assay system to test whether the combined usage of low concentrations of both inhibitor types would show synergistic effects on EGFR signaling. The cancer therapy using inhibitor couples is already in focus of research, because synergistic inhibition of cancer cell migration by using different inhibitor classes in lower doses could potentially reduce side effects in cancer therapies [8, 13].

**Fig 7:**
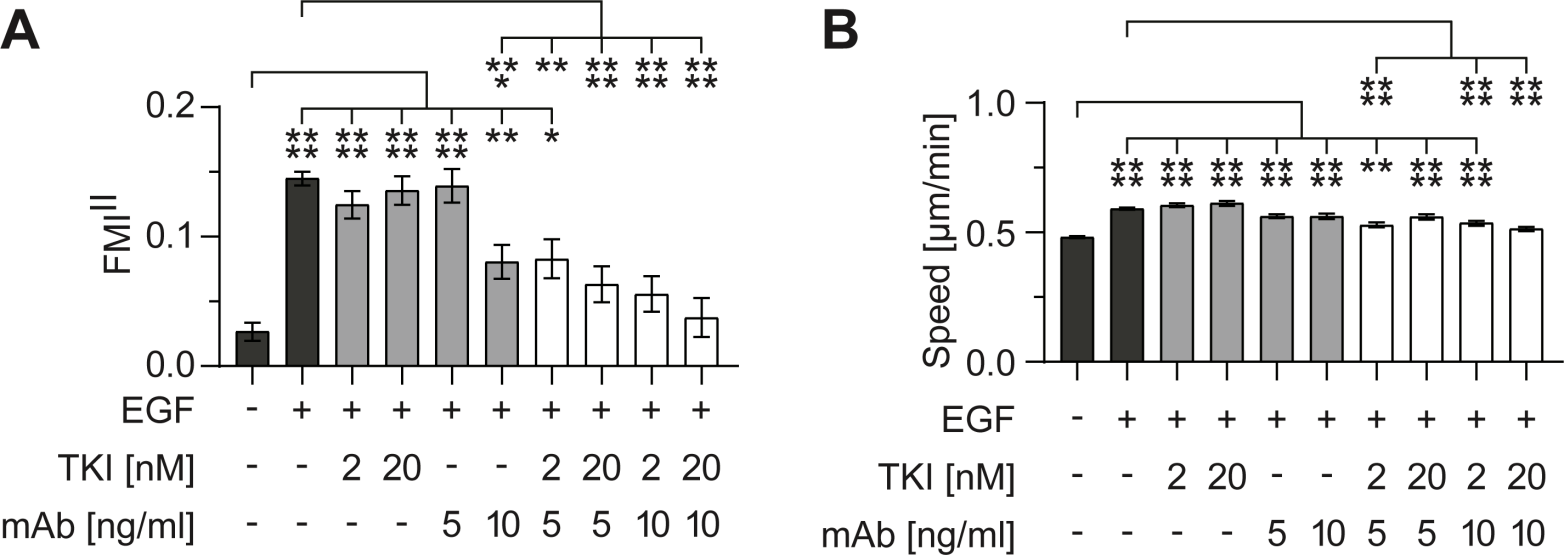
Synergistic inhibition of MDA-MB-231 cell migration in a 1.5 nM/mm EGF gradient. The effect of the combination of EGFR inhibitors AG1478 (as TKI) and monoclonal anti-EGFR antibody 225 (as mAb) on migration was evaluated by the (A) forward migration indices in direction of the gradient (FMI^II^) and (B) the cell speed. Four different combinations of AG1478 and anti-EGFR antibody were tested (white bars). Results were compared with the respective negative control without EGF gradient and gradient control without inhibitors (dark gray bars), as well as with both inhibitors applied separately (light gray bars). Significances are indicated by asterisks with * for 0.01<p<0.05, ** for 0.001<p<0.01, *** for 0.0001<p<0.001, and **** for p<0.0001.

To test for synergistic inhibition, we chose to combine two low, non-effective concentrations of AG1478 (TKI: 2 and 20 nM), with two low, non-effective concentrations of the monoclonal anti-EGFR antibody (mAb: 5 and 10 ng/ml) (Fig 7, light gray bars). As controls, experiments without EGF in the entire system and EGF gradient experiments without inhibitors were performed (Fig 7, dark gray bars). While being non-effective when applied separately, synergistic use of the TKI and the mAb led to a significant reduction of EGFR-mediated chemotaxis (Fig 7A). The chemokinetic response was also reduced, even at low inhibitor concentrations (Fig 7B). The combination of 20 nM TKI and 10 ng/ml mAb showed the highest inhibition effect for EGF-mediated chemotaxis, even though a tenfold higher dose of TKI and a fivefold higher dose of mAb were needed to show a comparable inhibition effect when used separately (Fig 6A, 200 nM; Fig 6C, 50 ng/ml).

The highest synergistic effect at low doses was shown at the combination of 2 nM TKI and 5 ng/ml mAb (Fig 7A). Separately, both concentrations did not inhibit EGF-mediated chemotaxis. Combined, both concentrations led to a significant reduction of chemotaxis.

EGFR is overexpressed, dysregulated, or mutated in many epithelial malignancies, making it a target of high promise in oncology [8–11]. mAbs have the advantage that they are highly selective to the receptor, because they recognize EGFR exclusively. However, mutated forms of EGFR have been identified in selected cancers where ligand-independent signaling occurs [11]. One major advantage of using TKIs over mAbs is their ability to inhibit cancer cells carrying the truncated forms of the receptor that are constitutively activated. Using inhibitor couples in cancer therapy exploits the advantages of both inhibiting mechanisms while reducing the applied inhibitor doses.

## Supporting information

S1 Fig Definitions in the 2D trajectory plots. The cell trajectories are extrapolated to (x,y) = 0, at time point 0 h (= slice 0). “I” is the index of different single cells. The first cell has the index “1”, the last one “n”. n = number of cells. x_i,end_, y_i,end_ = coordinates of cell end point, and d_i,accum_ = accumulated distance of cell path.
(TIF)

S2 Fig MDA-MB-231 cell migration in the presence of EGF in standard DMEM/F-12 medium with different serum concentrations in comparison with serum-free growth medium UC. Cell migration was analyzed by determining the cell speed of MDA-MB-231 cells migrating in pure medium (-/-), in a 1.5 nM/mm EGF gradient (EGF/-) and in 1.5 nM EGF in the entire chamber (EGF/EGF). Significances are indicated by asterisks with * for 0.01<p<0.05, ** for 0.001<p<0.01, *** for 0.0001<p<0.001, and **** for p<0.0001.
(TIF)

S3 Fig FMI analysis by manually tracked or simulated cell trajectories. 15 cell trajectories of manually tracked cells were randomly picked for each, (A) a 1.5 nM/mm EGF gradient (EGF/-) experiment and (B) a 1.5 nM EGF in the entire system (EGF/EGF) experiment. (C) A biased random walk and (D) a random walk were simulated and also 15 cell trajectories were illustrated, (E) The FMI^II^ values for random walk (indicated in red) and biased random walk (indicated in blue) were calculated and plotted against each step of the simulation.
(TIF)

S4 Fig MDA-MB-231 cell migration in the presence of EGF. Serum-free medium containing EGF in different concentrations (0.015–15 nM) was filled in the entire system of the chemotaxis chamber (EGF/EGF). Cell migration was analyzed by determining the cell speed. Significances are indicated by asterisks with * for 0.01<p<0.05, ** for 0.001<p<0.01, *** for 0.0001<p<0.001, and **** for p<0.0001.
(TIF)

S5 Fig MDA-MB-231 cell migration in linear EGF gradients. Serum-free medium UC containing EGF in different concentrations (0.015–15 nM) was filled in one reservoir and pure serum-free medium UC in the other reservoir (EGF/-). In the chemotaxis chamber (with a distance of –500 to 500 **μ**m from the center of the observation area), all tested stable concentration gradients shared the same signal-to-noise relation (▿c/c).
(TIF)
