# Supplementary figures and images for: Characterization of EGF-guided MDA-MB-231 cell chemotaxis in vitro using a physiological and highly sensitive assay system
Source: PLoS One. 2018 Sep 13;13(9):e0203040. doi: 10.1371/journal.pone.0203040 (PMC6136702; doi:10.1371/journal.pone.0203040)

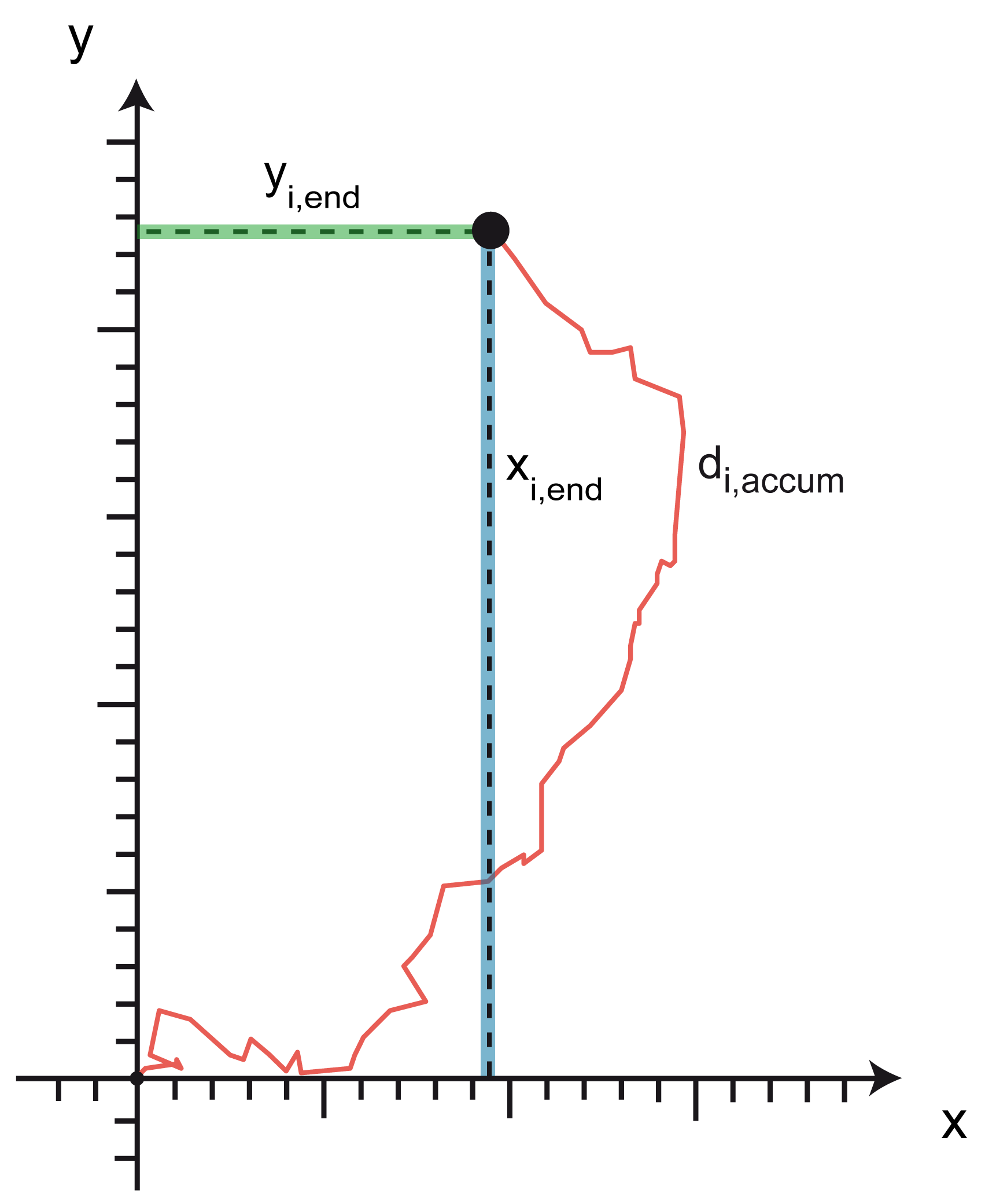

Supplement: S1 Fig — The cell trajectories are extrapolated to (x,y) = 0, at time point 0 h (= slice 0). “I” is the index of different single cells. The first cell has the index “1”, the last one “n”. n = number of cells. xi,end, yi,end = coordinates of cell end point, and di,accum = accumulated distance of cell path. (TIF) [file pone.0203040.s001.tif]

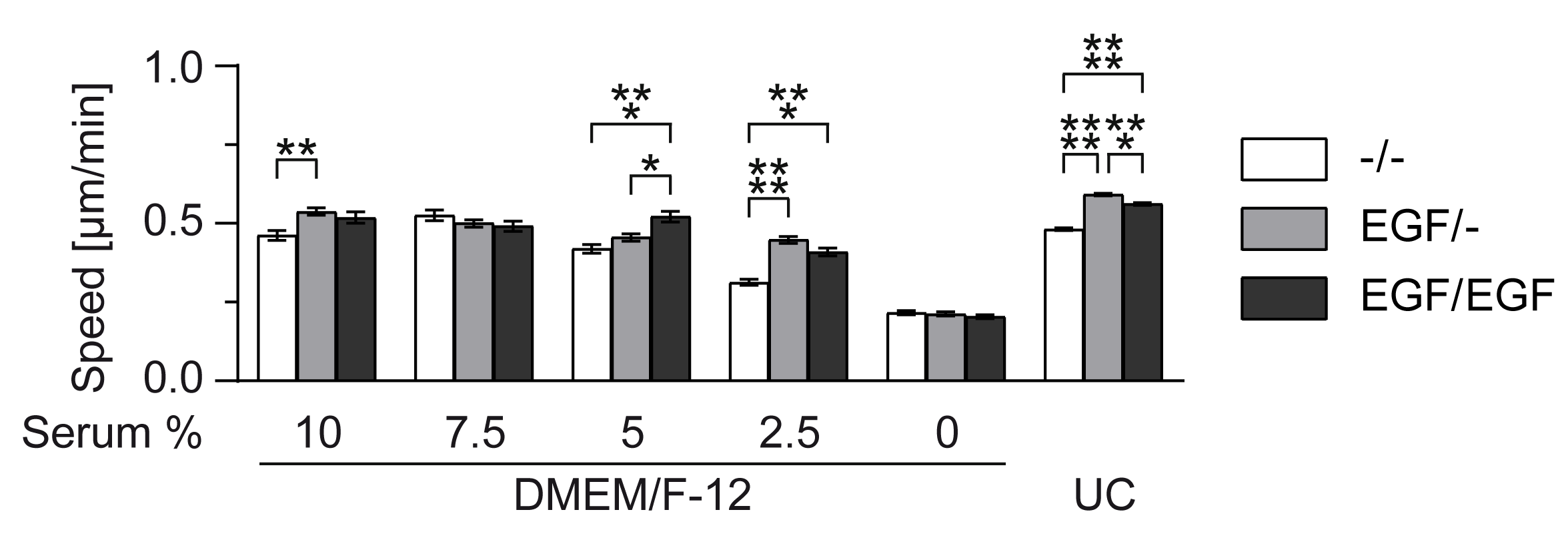

Supplement: S2 Fig — Cell migration was analyzed by determining the cell speed of MDA-MB-231 cells migrating in pure medium (-/-), in a 1.5 nM/mm EGF gradient (EGF/-) and in 1.5 nM EGF in the entire chamber (EGF/EGF). Significances are indicated by asterisks with * for 0.01<p<0.05, ** for 0.001<p<0.01, *** for 0.0001<p<0.001, and **** for p<0.0001. (TIF) [file pone.0203040.s002.tif]

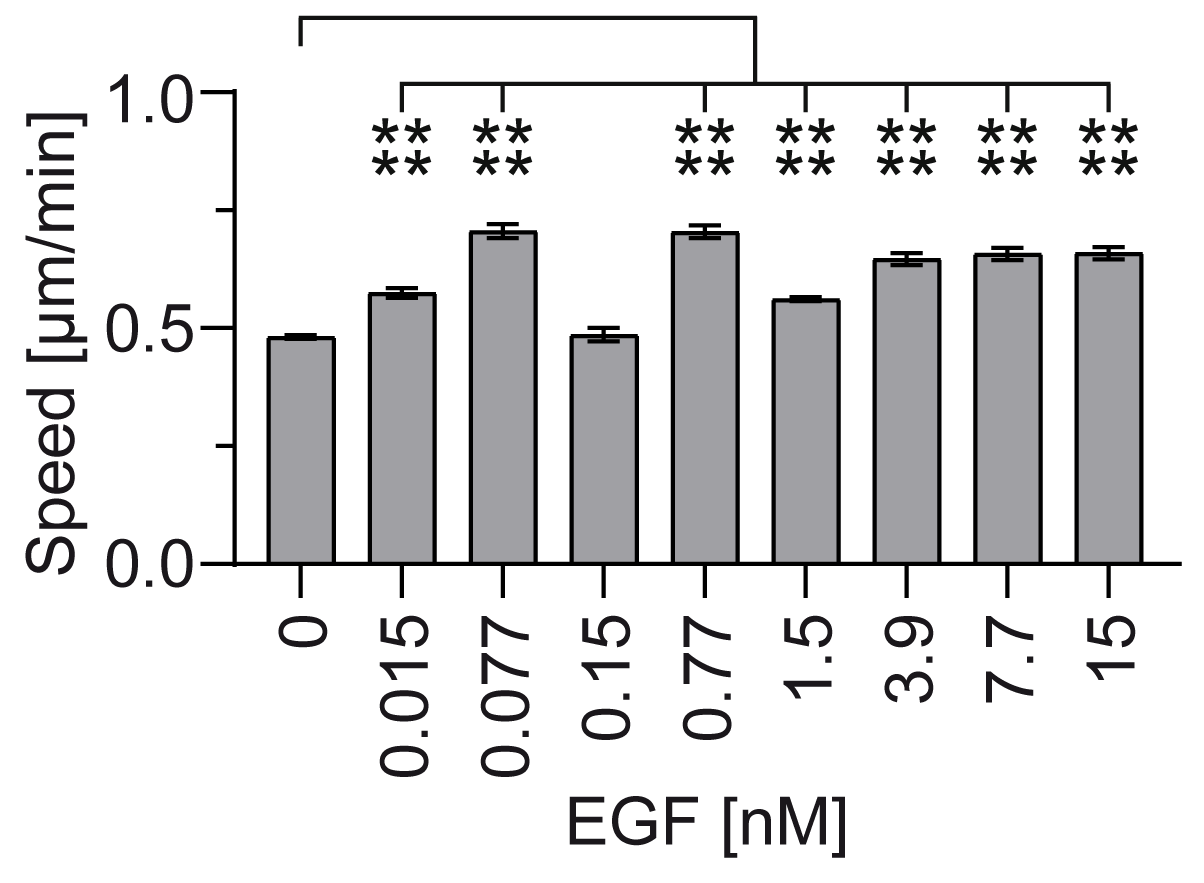

Supplement: S3 Fig — 15 cell trajectories of manually tracked cells were randomly picked for each, (A) a 1.5 nM/mm EGF gradient (EGF/-) experiment and (B) a 1.5 nM EGF in the entire system (EGF/EGF) experiment. (C) A biased random walk and (D) a random walk were simulated and also 15 cell trajectories were illustrated, (E) The FMIII values for random walk (indicated in red) and biased random walk (indicated in blue) were calculated and plotted against each step of the simulation. (TIF) [file pone.0203040.s003.tif]

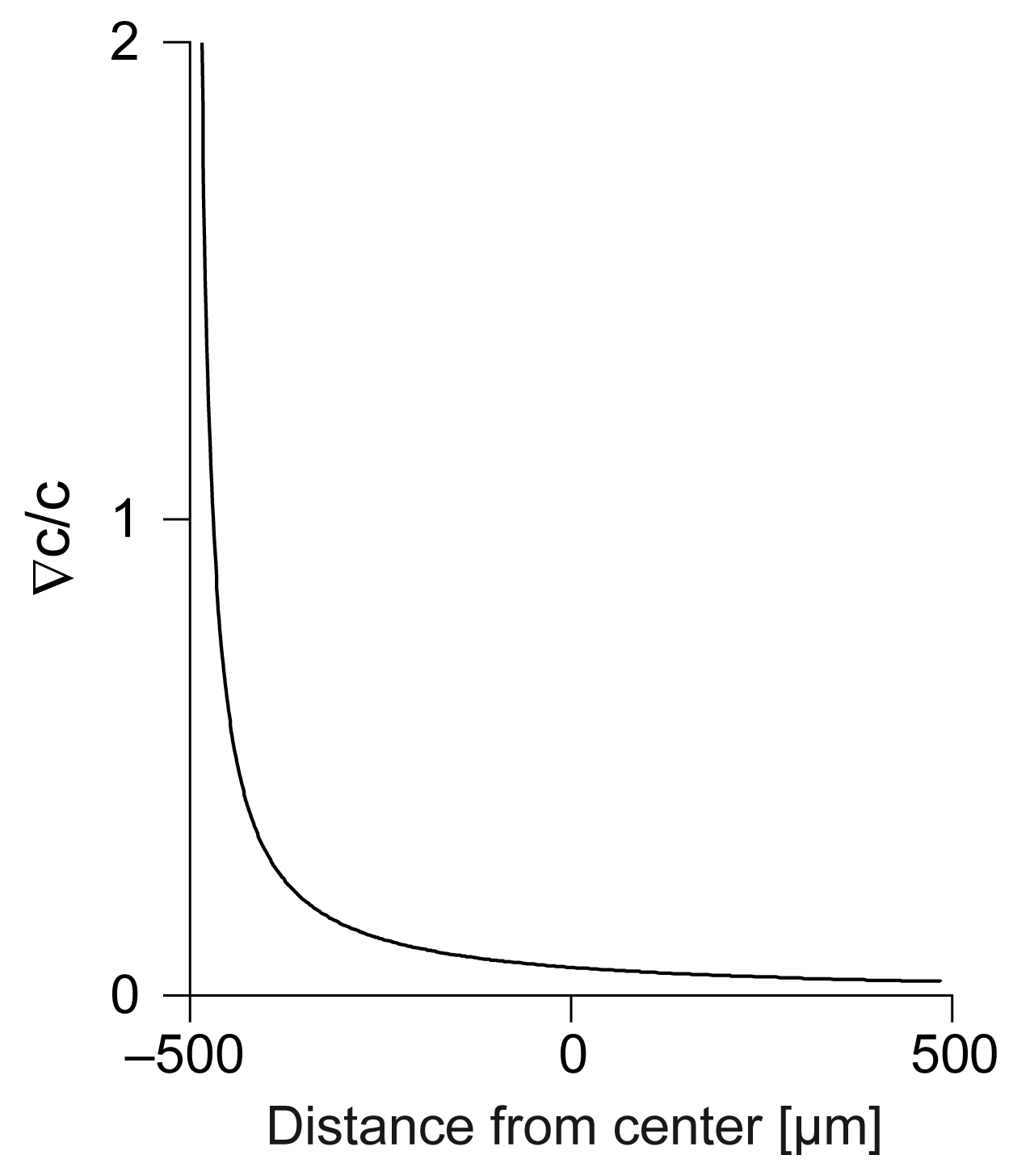

Supplement: S4 Fig — Serum-free medium containing EGF in different concentrations (0.015–15 nM) was filled in the entire system of the chemotaxis chamber (EGF/EGF). Cell migration was analyzed by determining the cell speed. Significances are indicated by asterisks with * for 0.01<p<0.05, ** for 0.001<p<0.01, *** for 0.0001<p<0.001, and **** for p<0.0001. (TIF) [file pone.0203040.s004.tif]

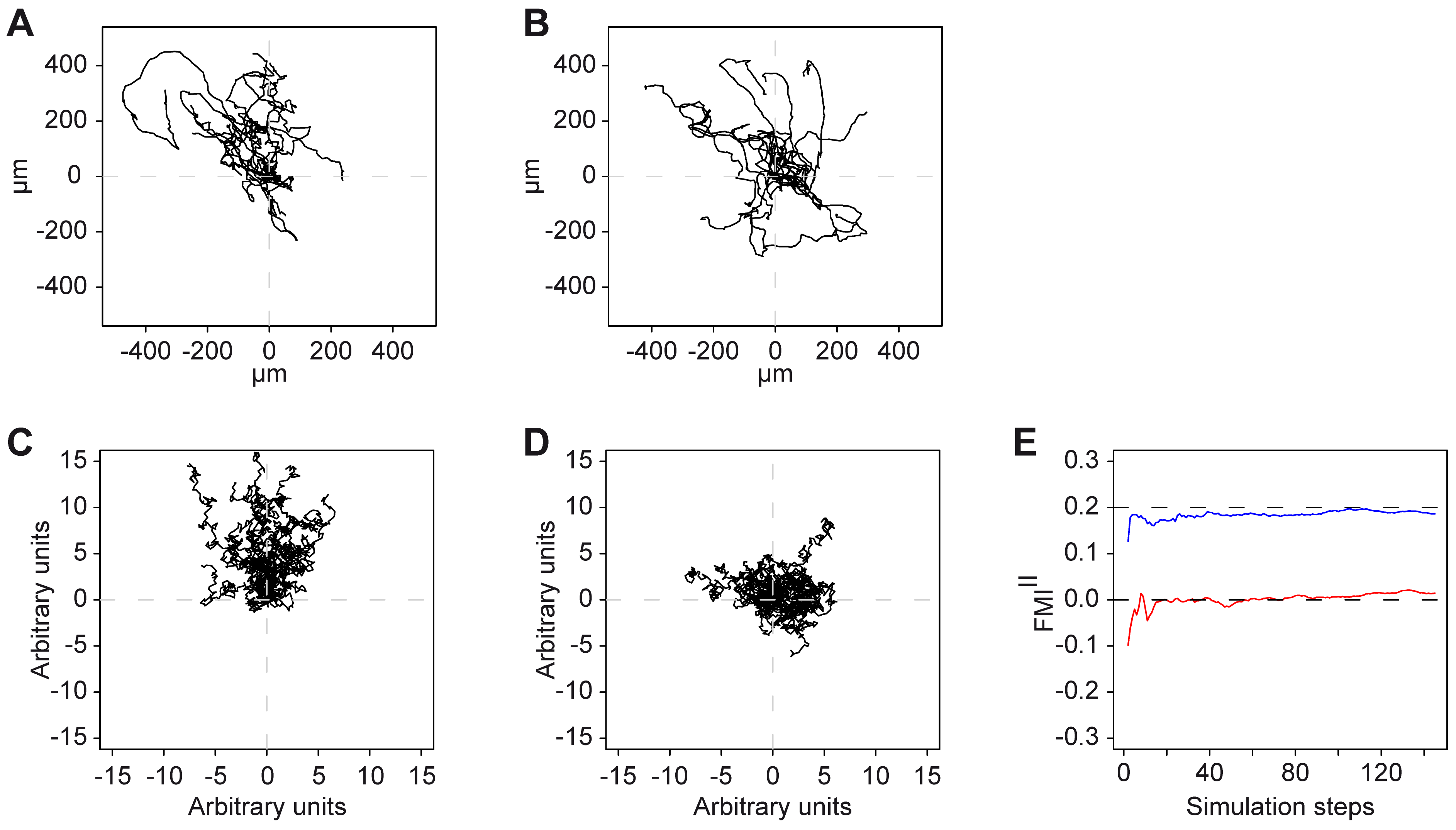

Supplement: S5 Fig — Serum-free medium UC containing EGF in different concentrations (0.015–15 nM) was filled in one reservoir and pure serum-free medium UC in the other reservoir (EGF/-). In the chemotaxis chamber (with a distance of –500 to 500 μm from the center of the observation area), all tested stable concentration gradients shared the same signal-to-noise relation (▿c/c). (TIF) [file pone.0203040.s005.tif]
